# Allergen immunotherapy in MASK‐air users in real‐life: Results of a Bayesian mixed‐effects model

**DOI:** 10.1002/clt2.12128

**Published:** 2022-03-28

**Authors:** Bernardo Sousa‐Pinto, Luís Filipe Azevedo, Ana Sá‐Sousa, Rafael José Vieira, Rita Amaral, Ludger Klimek, Wienczyslawa Czarlewski, Josep M. Anto, Anna Bedbrook, Violeta Kvedariene, Maria Teresa Ventura, Ignacio J. Ansotegui, Karl‐Christian Bergmann, Luisa Brussino, G. Walter Canonica, Victoria Cardona, Pedro Carreiro‐Martins, Thomas Casale, Lorenzo Cecchi, Tomás Chivato, Derek K. Chu, Cemal Cingi, Elisio M. Costa, Alvaro A. Cruz, Giulia De Feo, Philippe Devillier, Wytske J. Fokkens, Mina Gaga, Bilun Gemicioğlu, Tari Haahtela, Juan Carlos Ivancevich, Zhanat Ispayeva, Marek Jutel, Piotr Kuna, Igor Kaidashev, Helga Kraxner, Désirée E. Larenas‐Linnemann, Daniel Laune, Brian Lipworth, Renaud Louis, Michaël Makris, Riccardo Monti, Mario Morais‐Almeida, Ralph Mösges, Joaquim Mullol, Mikaëla Odemyr, Yoshitaka Okamoto, Nikolaos G. Papadopoulos, Vincenzo Patella, Nhân Pham‐Thi, Frederico S. Regateiro, Sietze Reitsma, Philip W. Rouadi, Boleslaw Samolinski, Milan Sova, Ana Todo‐Bom, Luis Taborda‐Barata, Peter Valentin Tomazic, Sanna Toppila‐Salmi, Joaquin Sastre, Ioanna Tsiligianni, Arunas Valiulis, Dana Wallace, Susan Waserman, Arzu Yorgancioglu, Mihaela Zidarn, Torsten Zuberbier, João Almeida Fonseca, Jean Bousquet, Oliver Pfaar

**Affiliations:** ^1^ MEDCIDS—Department of Community Medicine, Information and Health Decision Sciences, Faculty of Medicine, University of Porto Porto Portugal; ^2^ CINTESIS—Center for Health Technology and Services Research University of Porto Porto Portugal; ^3^ Department of Otolaryngology, Head and Neck Surgery Universitätsmedizin Mainz Mainz Germany; ^4^ Center for Rhinology and Allergology Wiesbaden Germany; ^5^ Medical Consulting Czarlewski Levallois France; ^6^ MASK‐air Montpellier France; ^7^ ISGlobal, Barcelona Institute for Global Health Barcelona Spain; ^8^ IMIM (Hospital del Mar Medical Research Institute) Barcelona Spain; ^9^ Universitat Pompeu Fabra (UPF) Barcelona Spain; ^10^ CIBER Epidemiología y Salud Pública (CIBERESP) Barcelona Spain; ^11^ Institute of Biomedical Sciences, Department of Pathology, Faculty of Medicine, Vilnius University Vilnius Lithuania; ^12^ Institute of Clinical Medicine, Clinic of Chest Diseases and Allergology, Faculty of Medicine, Vilnius University Vilnius Lithuania; ^13^ University of Bari Medical School Unit of Geriatric Immunoallergology Bari Italy; ^14^ Department of Allergy and Immunology Hospital Quirónsalud Bizkaia Bilbao Spain; ^15^ Institute of Allergology Charité—Universitätsmedizin Berlin Berlin Germany; ^16^ Corporate Member of Freie Universität Berlin Berlin Germany; ^17^ Humboldt‐Universität zu Berlin Berlin Germany; ^18^ Department of Medical Sciences Allergy and Clinical Immunology Unit University of Torino & Mauriziano Hospital Torino Italy; ^19^ Department of Biomedical Sciences Humanitas University Pieve Emanuele Italy; ^20^ Personalized Medicine, Asthma and Allergy Humanitas Clinical and Research Center IRCCS Rozzano Italy; ^21^ Allergy Section, Department of Internal Medicine, Hospital Vall d'Hebron & ARADyAL Research Network Barcelona Spain; ^22^ Serviço de Imunoalergologia, Hospital de Dona Estefânia, Centro Hospitalar Universitário de Lisboa Central Lisbon Portugal; ^23^ NOVA Medical School/Comprehensive Health Research Centre (CHRC) Lisbon Portugal; ^24^ Division of Allergy/immunology University of South Florida Tampa Florida USA; ^25^ SOS Allergology and Clinical Immunology, USL Toscana Centro Prato Italy; ^26^ School of Medicine University CEU San Pablo Madrid Spain; ^27^ Department of Medicine and Health Research Methods, Evidence & Impact, McMaster University Hamilton Ontario Canada; ^28^ Eskisehir Osmangazi University Medical Faculty ENT Department Eskisehir Turkey; ^29^ UCIBIO, REQUINTE, Faculty of Pharmacy and Competence Center on Active and Healthy Ageing of University of Porto (Porto4Ageing) Porto Portugal; ^30^ Fundaçao ProAR, Federal University of Bahia Salvador Bahia Brazil; ^31^ GARD/WHO Planning Group Salvador Bahia Brazil; ^32^ Department of Medicine, Surgery and Dentistry ‘Scuola Medica Salernitana’, University of Salerno Salerno Italy; ^33^ VIM Suresnes, UMR_0892, Pôle des Maladies des Voies Respiratoires, Hôpital Foch, Université Paris‐Saclay Suresnes France; ^34^ Department of Otorhinolaryngology Amsterdam University Medical Centres, location AMC Amsterdam The Netherlands; ^35^ ERS President 2017−2018, Athens Chest Hospital, 7th Respiratory Medicine Department and Asthma Center Athens Greece; ^36^ Department of Pulmonary Diseases Istanbul University‐Cerrahpasa Cerrahpasa Faculty of Medicine Istanbul Turkey; ^37^ Skin and Allergy Hospital Helsinki University Hospital University of Helsinki Helsinki Finland; ^38^ Servicio de Alergia e Immunologia, Clinica Santa Isabel Buenos Aires Argentina; ^39^ Department of Allergology and Clinical Immunology of the Kazakh National Medical University Almaty Kazakhstan; ^40^ Department of Clinical Immunology Wrocław Medical University Wroclaw Poland; ^41^ ALL‐MED Medical Research Institute Wroclaw Poland; ^42^ Division of Internal Medicine, Asthma and Allergy, Barlicki University Hospital, Medical University of Lodz Lodz Poland; ^43^ Poltava State Medical University Poltava Ukraine; ^44^ Department of Otorhinolaryngology, Head and Neck Surgery, Semmelweis University Budapest Hungary; ^45^ Center of Excellence in Asthma and Allergy Médica Sur Clinical Foundation and Hospital México City Mexico; ^46^ KYomed INNOV Montpellier France; ^47^ Scottish Centre for Respiratory Research, Cardiovascular & Diabetes Medicine, Medical Research Institute, Ninewells Hospital, University of Dundee Dundee UK; ^48^ Department of Pulmonary Medicine CHU Sart‐Tilman Liege Belgium; ^49^ GIGA I3 Research Group Liege Belgium; ^50^ Allergy Unit ‘D Kalogeromitros’, 2nd Department of Dermatology and Venereology, National & Kapodistrian University of Athens, ‘Attikon’ University Hospital Chaidari Greece; ^51^ Department of Cardiovascular and Thoracic Sciences Fondazione Policlinico Universitario A Gemelli IRCCS Università Cattolica del Sacro Cuore Rome Italy; ^52^ Allergy Center CUF Descobertas Hospital Lisbon Portugal; ^53^ ClinCompetence Cologne GmbH Cologne Germany; ^54^ IMSB, Medical Faculty, University of Cologne Cologne Germany; ^55^ Rhinology Unit & Smell Clinic, ENT Department, Hospital Clínic Barcelona Spain; ^56^ Clinical & Experimental Respiratory Immunoallergy, IDIBAPS, CIBERES, University of Barcelona Barcelona Spain; ^57^ EFA European Federation of Allergy and Airways Diseases Patients' Associations Brussels Belgium; ^58^ Department of Otorhinolaryngology Chiba University Hospital Chiba Japan; ^59^ Allergy Department, 2nd Pediatric Clinic, University of Athens Athens Greece; ^60^ Division of Allergy and Clinical Immunology, Department of Medicine, Agency of Health ASL Salerno, ‘Santa Maria della Speranza’ Hospital Salerno Italy; ^61^ Ecole Polytechnique Palaiseau, IRBA (Institut de Recherche bio‐Médicale des Armées) Bretigny France; ^62^ Department of Otolaryngology‐Head and Neck Surgery, Eye and Ear University Hospital Beirut Lebanon; ^63^ ENT Department Dar Al Shifa Hospital Salmiya Kuwait; ^64^ Department of Prevention of Environmental Hazards, Allergology and Immunology, Medical University of Warsaw Warsaw Poland; ^65^ Department of Pulmonary Medicine and Tuberculosis University Hospital Brno Liskovec Czech Republic; ^66^ Faculty of Health Sciences University of Beira Interior Covilhã Portugal; ^67^ UBIAir—Clinical & Experimental Lung Centre University of Beira Interior Covilhã Portugal; ^68^ Department of Immunoallergology Cova da Beira University Hospital Centre Covilhã Portugal; ^69^ Department of General ORL, H&NS, Medical University of Graz, ENT‐University Hospital Graz Graz Austria; ^70^ Fundacion Jimenez Diaz, CIBERES, Faculty of Medicine, Autonoma University of Madrid Madrid Spain; ^71^ Health Planning Unit, Department of Social Medicine, Faculty of Medicine, University of Crete Rethymno Greece; ^72^ International Primary Care Respiratory Group IPCRG Aberdeen Scotland; ^73^ Institute of Clinical Medicine and Institute of Health Sciences Medical Faculty of Vilnius University Vilnius Lithuania; ^74^ Nova Southeastern University Fort Lauderdale Florida USA; ^75^ Department of Medicine, Clinical Immunology and Allergy, McMaster University Hamilton Ontario Canada; ^76^ Department of Pulmonology Celal Bayar University Manisa Turkey; ^77^ University Clinic of Respiratory and Allergic Diseases Golnik Slovenia; ^78^ University of Ljubljana Faculty of Medicine Ljubljana Slovenia; ^79^ Fraunhofer Institute for Translational Medicine and Pharmacology ITMP, Allergology and Immunology Berlin Germany; ^80^ Medicina, EDucação, I&D e Avaliação, Lda (MEDIDA) Porto Portugal; ^81^ University Hospital Montpellier Montpellier France; ^82^ Department of Otorhinolaryngology, Head and Neck Surgery, Section of Rhinology and Allergy University Hospital Marburg, Philipps‐Universität Marburg Marburg Germany

**Keywords:** allergic rhinitis, immunotherapy, mobile health, patient‐reported outcomes, real‐life data analysis

## Abstract

**Background:**

Evidence regarding the effectiveness of allergen immunotherapy (AIT) on allergic rhinitis has been provided mostly by randomised controlled trials, with little data from real‐life studies.

**Objective:**

To compare the reported control of allergic rhinitis symptoms in three groups of users of the MASK‐air^®^ app: those receiving sublingual AIT (SLIT), those receiving subcutaneous AIT (SCIT), and those receiving no AIT.

**Methods:**

We assessed the MASK‐air^®^ data of European users with self‐reported grass pollen allergy, comparing the data reported by patients receiving SLIT, SCIT and no AIT. Outcome variables included the daily impact of allergy symptoms globally and on work (measured by visual analogue scales—VASs), and a combined symptom‐medication score (CSMS). We applied Bayesian mixed‐effects models, with clustering by patient, country and pollen season.

**Results:**

We analysed a total of 42,756 days from 1,093 grass allergy patients, including 18,479 days of users under AIT. Compared to no AIT, SCIT was associated with similar VAS levels and CSMS. Compared to no AIT, SLIT‐tablet was associated with lower values of VAS global allergy symptoms (average difference = 7.5 units out of 100; 95% credible interval [95%CrI] = −12.1;−2.8), lower VAS Work (average difference = 5.0; 95%CrI = −8.5;−1.5), and a lower CSMS (average difference = 3.7; 95%CrI = −9.3;2.2). When compared to SCIT, SLIT‐tablet was associated with lower VAS global allergy symptoms (average difference = 10.2; 95%CrI = −17.2;−2.8), lower VAS Work (average difference = 7.8; 95%CrI = −15.1;0.2), and a lower CSMS (average difference = 9.3; 95%CrI = −18.5;0.2).

**Conclusion:**

In patients with grass pollen allergy, SLIT‐tablet, when compared to no AIT and to SCIT, is associated with lower reported symptom severity. Future longitudinal studies following internationally‐harmonised standards for performing and reporting real‐world data in AIT are needed to better understand its ‘real‐world’ effectiveness.

## 1 INTRODUCTION

Allergen immunotherapy (AIT) is an effective treatment for allergic rhinitis and/or asthma, as demonstrated by large well‐designed randomised controlled trials (RCTs).^1^, ^2^, ^3^ Such RCTs have been carried out with large studies on sublingual immunotherapy (SLIT)^1^, ^2^ and with smaller ones on subcutaneous immunotherapy (SCIT).^3^, ^4^ Based on the available evidence for both application routes,^5^ several guidelines with clinical evidence‐based recommendations have recently been published by the European Academy of Allergy and Clinical Immunology^6^ and Allergic Rhinitis and its Impact on Asthma (ARIA; an expert consortium issuing recommendations based on a GRADE evaluation).^7^, ^8^, ^9^, ^10^

While RCTs are requested for market authorisation purposes, following the formal regulation by authorities such as the European Medicines Agency, they narrow the study population based on specific criteria as pre‐defined in study protocols.^11^ It is unclear as to whether the effects of treatments seen in highly‐controlled RCTs are similar to those in less‐controlled pragmatic study designs, such as large observational studies (often referred to as ‘real‐world data’ [RWD]).^11^

Results from clinical trials should therefore be complemented with those from RWD, which can be obtained using data from electronic health records or from monitoring tools such as mobile apps. Several retrospective studies in administrative databases have suggested the efficacy of AIT in rhinitis and asthma.^12^, ^13^ Evidence from mobile apps is more scarce, but RWD obtained from mobile apps is an increasing and demanding field, not only in the allergy domain, but also in several other chronic diseases, including different conditions such as sleep disturbances,^14^ rheumatologic diseases^15^ or diabetes.^16^ This reflects the high potential of mobile apps for scientific purposes, patient self‐management and/or adherence, as well as the encouraging results some apps have displayed in improving adherence and/or clinical trials.^17^ A recent proof‐of‐concept study clearly demonstrated that MASK‐air^®^ (a mobile app with a monitoring questionnaire assessing the impact of allergic symptoms, work and medication use each day^18^, ^19^) is a valuable tool for assessing the impact of AIT.^20^ This first analysis revealed that days under AIT are associated with approximately a 25% improved control of allergic rhinitis symptoms. Interestingly, the same magnitude of effect was observed when comparing days without symptomatic treatment versus those under monotherapy and those under co‐medication.^20^ However, this study did not compare the different application routes of AIT and the treatment schedules, neither did it take into account the different countries or pollen seasons.

Therefore, the aim of the present study was to use MASK‐air^®^ RWD to compare the reported control of allergic rhinitis symptoms in SCIT, SLIT and no AIT users allergic to grass pollen.

## 2 METHODS

### 2.1 Study design

This is a cross‐sectional study using MASK‐air^®^ data. We compared SCIT, SLIT‐tablet and no AIT for the severity of reported allergic rhinitis symptoms, their impact on work and a combined symptom‐medication score (CSMS). We took into account the differences across users, countries and seasons, by performing analyses in which the observations were clustered by user, country and season.

### 2.2 Setting

MASK‐air^®^ was initiated in 2015 and is available in 27 countries (www.mask‐air.com).^21^, ^22^ For each AIT‐specific item, we included data from all MASK‐air^®^ European countries with at least 150 days of reporting.

### 2.3 Participants

We included users aged 16–90 years, who reported allergic rhinitis and allergy to grass pollen.^21^ In the app, they reported whether or not they were under AIT (SCIT/SLIT). All analysed data concerned the period 21 May 2015 to 6 December 2020.

### 2.4 Ethics

MASK‐air^®^ is CE1 registered and follows the General Data Protection Regulation.^23^ An independent review board approval was not required for this specific study as it is an observational study. All data were anonymised prior to the study (including geolocation‐related data) using k‐anonymity, and users agreed to the analysis of their data in the terms of use (translated into all languages and customised according to the legislation of each country, allowing the use of the results for research purposes).

### 2.5 Data sources and variables

MASK‐air^®^ comprises a daily monitoring questionnaire which assesses the impact of allergic rhinitis using visual analogue scales (VASs) on a 0–100 scale, which display high intra‐rater validity and moderate‐high validity, test‐retest reliability and responsiveness (Supplementary Table S2).^24^ The questionnaire includes a question on how much overall allergic rhinitis symptoms are bothering the user on that day (‘VAS Global Allergy Symptoms’), as well as one on how much allergic symptoms are affecting work on that day (‘VAS Work’; only presented if the user reports to be working on that day). In addition to the VASs, the MASK‐air^®^ daily monitoring questionnaire asks users whether they took medication or had been under AIT on that day. In the configuration of their profile, MASK‐air^®^ users can provide information on their age, sex, country, allergen sensitisation, allergy symptoms, smoking status, and—if under AIT—type of AIT (SCIT or SLIT, including SLIT‐tablet).

When responding to the MASK‐air^®^ daily monitoring questionnaire, it is not possible to skip any of the questions, and data are saved to the dataset only after the final answer. This precludes any missing data.

### 2.6 Size of the study

For each specific AIT type, we analysed all of the data available from European countries with at least 150 days of use/observations.

### 2.7 Biases

There are potential information biases related to the self‐reported nature of the data collection. Potential selection bias might be introduced due to the fact that app users are not representative of all patients with rhinitis.

### 2.8 Data analysis

Categorical variables were described using absolute and relative frequencies, while continuous variables were described using medians and interquartile ranges. In MASK‐air^®^, each reporting day corresponds to an observation. We compared the days of patients under SCIT, SLIT‐tablet and no AIT. The days of these different groups of patients were compared using VAS Global Allergy Symptoms, VAS Work, and a CSMS^37^ (mixed hypothesis‐ and data‐driven score calculated by multiplying VAS Global Allergy Symptoms by a medication factor; Supplementary Table S1).

To perform such comparisons, we applied three hierarchical models (also called ‘multilevel models’ or ‘mixed‐effects models’)—one for each score. For each model, the type of grass AIT was a fixed effect, while random effects included identification of the user (nested within the respective country) and indication as to whether the observation occurred within or outside the grass pollen season (we used Bedard's method to assess the grass pollen season^25^). In other words, we modelled the association between VAS and AIT type, taking into account the clustering of observations by users, by countries and by seasons (i.e., we adjusted our comparisons according to the clustering of multiple users' observations, of the user's country, and of whether the observation occurred within or outside the pollen season). Additional hierarchical models were built, which also adjusted for the patients' sex, age and comorbidities (asthma and conjunctivitis). Sensitivity analyses were performed with results stratified (i) by days during or outside the pollen season, and (ii) by countries with a higher number of observations under SCIT than under SLIT‐tablet versus countries with a higher number of observations under SLIT‐tablet than under SCIT.

Hierarchical models were applied using Bayesian methods. We opted for Bayesian approaches as they yield probability distributions of the parameters of interest (posterior probabilities) based on prior probability distributions and on the observed data.^26^ That is, in this study, for each comparison, we obtained the posterior probability distribution for the average difference of VAS, retrieving the mean value and the respective 95% credible interval (CrI; range of values within which, with 95% probability, the true VAS difference lies. In Bayesian statistics, uncertainty is expressed through CrI and not through classical confidence intervals or *p*‐values). This is a methodological advantage as it informs us of the probability of each AIT type being associated with a lower VAS or CSMS, besides allowing for the obtention of a probability distribution that can be graphically plotted. Uninformative prior distributions of dnorm[0,0.0001] and dunif[0,100] were respectively used for the regression coefficients and for the precision parameters.

All statistical analyses were performed using the software R (version 4.0.0.) with the rjags package. For each analysis, we ran 70,000 iterations with a burn‐in of 30,000 sample iterations.

## 3 RESULTS

### 3.1 Characteristics of the patients

We analysed 42,756 days from 1,093 grass allergy patients in 10 countries (Austria, France, Germany, Greece, Italy, Lithuania, Poland, Portugal, Spain and Switzerland). Of those 42,756 days, 18,479 (43.2%) were from users under AIT, including 12,675 days under SCIT (68.6% of AIT days) and 5,804 under SLIT‐tablet (31.4% of AIT days). SLIT‐drop was not analysed due to the low number of observations. The demographic and clinical characteristics of the patients are given in Table 1. The mean number of days reported per user is 38 for both no AIT and SCIT, and 47 for SLIT‐tablet.

**TABLE 1:** Demographic and clinical characteristics of assessed MASK‐air^®^ observations/days and respective users

	Immunotherapy			No AIT
	All	SCIT	SLIT‐tablet	
All observations/days—*N* [*N* users]	18,479 [457]	12,675 [334]	5804 [123]	24,277 [636]
Females—*N* (%) [*N* users (%)]	9367 (50.7) [225 (49.2)]	7065 (55.7) [169 (47.9)]	2302 (39.7) [56 (45.5)]	11,822 (48.7) [350 (55.0)]
Age—median (IQR)	34 (18)	34 (17)	34 (18)	40 (19)
Asthma—*N* (%) [*N* users (%)]	5114 (27.7) [162 (35.4)]	3598 (28.4) [126 (37.7)]	1516 (26.1) [36 (29.3)]	12,941 (53.3) [282 (44.3)]
VAS global allergy symptoms—median (IQR)	6 (18)	8 (20)	7 (19)	10 (22)
First day VAS—median (IQR)	27 (48)	30 (47)	18 (47)	32 (50)
VAS asthma—median (IQR)	0 (3)	0 (3)	0 (0)	2 (11)
First day VAS asthma—median (IQR)	0 (15)	0 (29)	0 (1)	3 (20)
Conjunctivitis—*N* (%) [*N* users (%)]	15,586 (84.3) [385 (84.2)]	10,096 (79.7) [276 (82.6)]	5490 (94.6) [109 (88.6)]	21,308 (87.8) [518 (81.4)]
VAS eyes symptoms—median (IQR)	1 (13)	1 (13)	1 (12)	5 (16)
First day VAS eyes—median (IQR)	8 (36)	8 (39)	7 (28)	9 (34)
VAS work—median (IQR)	2 (12)	4 (15)	1 (13)	7 (18)
First day VAS work—median (IQR)	12 (31)	15 (33)	10 (23)	17 (30)
Medications used—*N* (%) [*N* users (%)]	6791 (36.7) [300 (65.6)]	4733 (37.3) [232 (69.5)]	2058 (35.5) [68 (55.3)]	11,868 (48.9) [450 (70.8)]
Intranasal or ocular antihistamines	1632 (8.8) [75 (16.4)]	1008 (8.0) [54 (16.2)]	624 (10.8) [21 (17.1)]	2832 (11.7) [144 (22.6)]
Oral antihistamines	5222 (28.3) [266 (58.2)]	3707 (29.2) [204 (61.1)]	1515 (26.1) [62 (50.4)]	7742 (31.9) [382 (60.1)]
Intranasal steroids	3102 (16.8) [145 (31.7)]	2521 (19.9) [119 (35.6)]	581 (10.0) [26 (21.1)]	5670 (23.4) [250 (39.3)]
Oral steroids	79 (0.4) [12 (2.6)]	64 (0.5) [7 (2.1)]	15 (0.3) [5 (4.1)]	214 (0.9) [26 (4.1)]
Other rhinitis medications	538 (2.9) [61 (13.3)]	429 (3.4) [47 (14.1)]	109 (1.9) [14 (11.4)]	2073 (8.5) [98 (15.4)]
Grass pollen season—*N* (%)	4471 (24.2)	3146 (24.8)	1325 (22.8)	5294 (21.8)

Abbreviations: AIT, allergen immunotherapy; IQR, interquartile range; SCIT, subcutaneous immunotherapy; SLIT‐tablet, sublingual AIT exclusively by tablets; VAS, visual analogue scale.

### 3.2 Major results

Overall, patients receiving AIT had a lower median VAS Global allergy symptoms than patients under no AIT (6 vs. 10, Table 2A), a lower median VAS Work (2 vs. 7, Table 2C), and a lower median CSMS (8 vs. 11, Table 2C).

**TABLE 2:** Number of MASK‐air^®^ reporting days/observations and associated allergic rhinitis symptoms and their impact on work under each grass immunotherapy (allergen immunotherapy [AIT]) type

A. Global allergy symptom control								
Medication scheme	*N* observations/days (*N* users)				VAS global allergy symptoms—median (IQR)			
	Immunotherapy			No AIT	Immunotherapy			No AIT
	All	SCIT	SLIT‐tablet		All	SCIT	SLIT‐tablet	
All countries	18,479 (457)	12,675 (334)	5804 (123)	24,277 (636)	6 (18)	8 (20)	7 (19)	10 (22)
Austria	626 (20)	626 (20)	‐^a^	698 (39)	9 (23)	9 (23)	‐^a^	8 (17)
France	331 (35)	‐^a^	331 (35)	2117 (65)	7 (23)	‐^a^	15 (33)	7 (20)
Germany	4048 (91)	3219 (80)	829 (11)	3917 (112)	12 (19)	13 (20)	9 (18)	16 (26)
Greece	910 (14)	910 (14)	‐^a^	767 (13)	7 (18)	7 (18)	‐	0 (13)
Italy	5808 (84)	1671 (15)	4137 (69)	4193 (115)	6 (16)	6 (12)	6 (18)	9 (21)
Lithuania	1840 (15)	1840 (15)	‐^a^	3818 (72)	0 (11)	0 (18)	‐^a^	5 (14)
Poland	2033 (86)	2033 (86)	‐^a^	2691 (84)	4 (16)	4 (16)	‐^a^	11 (24)
Portugal	687 (35)	527 (32)	160 (3)	1192 (44)	13 (30)	13 (30)	10 (18)	19 (21)
Spain	993 (43)	993 (43)	‐^a^	4477 (71)	7 (23)	13 (27)	‐^a^	11 (22)
Switzerland	1203 (34)	856 (29)	347 (5)	407 (21)	8 (19)	9 (16)	0 (14)	14 (24)

B. Impact of allergic rhinitis symptoms on work								
Medication scheme	*N* observations/days (*N* users)				VAS work—median (IQR)			
	Immunotherapy			No AIT	Immunotherapy			No AIT
	All	SCIT	SLIT‐tablet		All	SCIT	SLIT‐tablet	
All countries	9614 (347)	6241 (259)	3373 (88)	11,756 (485)	2 (12)	4 (15)	1 (13)	7 (18)
Austria	353 (15)	353 (15)	‐^a^	302 (28)	5 (18)	5 (18)	‐^a^	4 (15)
France	190 (28)	‐^a^	190 (28)	1060 (52)	4 (18)	‐^a^	5 (20)	1 (10)
Germany	1745 (74)	1472 (65)	273 (9)	2085 (86)	7 (16)	8 (18)	0 (9)	12 (21)
Greece	380 (11)	380 (11)	‐^a^	411 (10)	12 (6)	12 (16)	‐^a^	0 (6)
Italy	3708 (56)	955 (9)	2753 (47)	2120 (84)	1 (11)	0 (7)	1 (12)	8 (17)
Lithuania	1042 (11)	1042 (11)	‐^a^	1766 (57)	0 (7)	0 (10)	‐^a^	3 (11)
Poland	926 (63)	926 (63)	‐^a^	1235 (62)	2 (12)	2 (12)	‐^a^	11 (18)
Portugal	313 (24)	313 (24)	‐^a^	499 (37)	4 (17)	4 (17)	‐^a^	13 (18)
Spain	371 (35)	371 (35)	‐^a^	2044 (52)	10 (29)	10 (29)	‐^a^	7 (19)
Switzerland	586 (30)	429 (26)	157 (4)	234 (17)	0 (17)	2 (18)	0 (16)	6 (16)

C. Combined symptom‐medication score								
Medication scheme	*N* observations/days (*N* users)				Combined symptom‐medication score—median (IQR)			
	Immunotherapy			No AIT	Immunotherapy			No AIT
	All	SCIT	SLIT‐tablet		All	SCIT	SLIT‐tablet	
All countries	16,129 (310)	11,141 (240)	4988 (70)	21,567 (462)	8.0 (21.0)	8.8 (22.0)	12.0 (23.3)	11.0 (23.1)
Austria	538 (12)	538 (12)	‐^a^	620 (26)	12.0 (27.0)	12.0 (27.0)	‐	7.6 (17.0)
France	275 (19)	‐^a^	275 (19)	1880 (50)	19.8 (35.7)	‐^a^	19.8 (35.7)	9.0 (25.3)
Germany	3391 (60)	2563 (50)	828 (10)	2730 (67)	12.0 (20.0)	12.1 (21.3)	9.4 (19.0)	16.0 (25.0)
Greece	904 (12)	904 (12)	‐^a^	705 (10)	7.0 (19.8)	7.0 (19.8)	‐^a^	0 (13.0)
Italy	5203 (47)	1661 (9)	3542 (38)	3922 (83)	6.6 (17.6)	7.0 (13.0)	6.2 (20.8)	10.0 (22.7)
Lithuania	1816 (13)	1816 (13)	‐^a^	3696 (56)	0 (18.8)	0 (18.8)	‐^a^	6.0 (16.0)
Poland	1729 (62)	1729 (62)	‐^a^	2596 (63)	5.2 (18.7)	5.2 (18.7)	‐^a^	13.2 (27.5)
Portugal	493 (26)	493 (26)	‐^a^	1035 (35)	13.0 (33.0)	13.0 (33.0)	‐^a^	19.0 (21.8)
Spain	729 (34)	729 (34)	‐^a^	3997 (56)	16.0 (31.4)	16.0 (31.4)	‐^a^	12.0 (24.8)
Switzerland	1051 (25)	708 (22)	343 (3)	386 (16)	9.0 (21.0)	10.4 (17.9)	0 (15.2)	13.0 (22.3)

Abbreviations: IQR, interquartile range; SCIT, subcutaneous immunotherapy; SLIT‐tablet, sublingual immunotherapy exclusively by tablets; VAS global allergy symptoms, visual analogue scale assessing the overall impact of allergic rhinitis symptoms on the user on that day; VAS work, visual analogue scale assessing the impact of allergic rhinitis symptoms on working activity of the user on that day.

a Number of observations/reporting days <150, precluding analysis.

**FIGURE 1:**
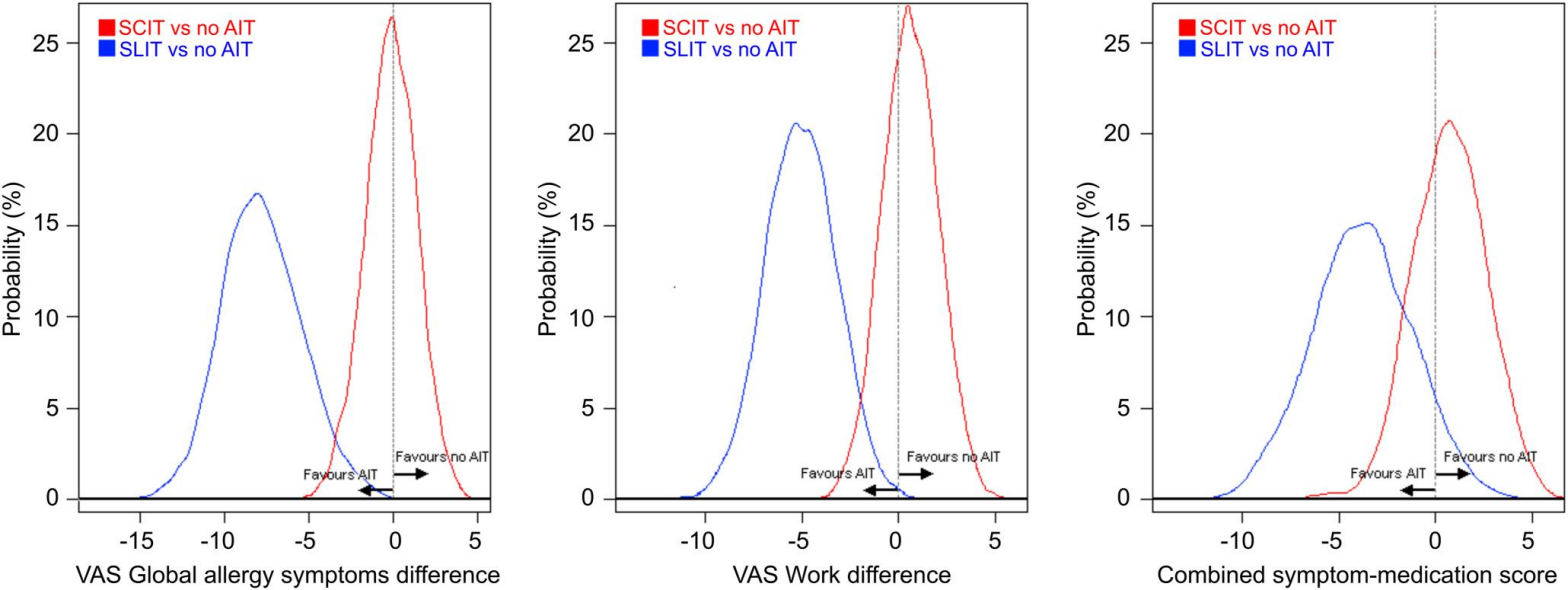
Probability distributions of the comparisons between grass subcutaneous immunotherapy (SCIT) versus no allergen immunotherapy (AIT), and grass sublingual immunotherapy (SLIT) by tablet versus no AIT

By comparison with no AIT, SCIT was associated with similar VAS Global allergy symptom levels (average difference = 0.2 units out of 100, 95%CrI = −3.2;2.8), VAS Work levels (0.6, −2.2;3.4), and CSMS values (0.8, −2.9;4.4). Overall, the probability of SCIT being better than no AIT was 55% for VAS Global allergy symptoms, 34% for VAS Work, and 33% for CSMS (Table 3; Figure 1).

**TABLE 3:** Results of the comparisons between different grass immunotherapy (allergen immunotherapy [AIT]) types

	Difference in VAS global allergy symptoms	Difference in VAS work	Difference in CSMS
A. Hierarchical models adjusting for the season, country and patient			
SCIT versus no AIT—Mean (CrI) [probability of SCIT being better than no AIT]	−0.2 (−3.2;2.8) [55%]	0.6 (−2.2;3.4) [34%]	0.8 (−2.9;4.4) [33%]
SLIT‐tablet versus no AIT—Mean (CrI) [probability of SLIT‐tablet being better than no AIT]	−7.5 (−12.1;−2.8) [99%]	−5.0 (−8.5;−1.5) [99%]	−3.7 (−9.3;2.2) [89%]
SLIT‐tablet versus SCIT—Mean (CrI) [probability of SLIT‐tablet being better than SCIT]	−10.2 (−17.5;−2.8) [99%]	−7.8 (−15.1;0.2) [97%]	−9.3 (−18.5;0.2) [97%]

B. Hierarchical models adjusting for the season, country, patient and his/her characteristics (sex, age and comorbidities)			
SCIT versus no AIT—Mean (CrI) [probability of SCIT being better than no AIT]	0.2 (−2.7;3.6) [47%]	1.1 (−1.6;3.8) [20%]	1.3 (−2.5;4.9) [25%]
SLIT‐tablet versus no AIT—Mean (CrI) [probability of SLIT‐tablet being better than no AIT]	−7.9 (−12.6;−3.5) [99%]	−4.8 (−8.4;1.1) [99%]	−2.1 (−8.0;3.5) [75%]
SLIT‐tablet versus SCIT—Mean (CrI) [probability of SLIT‐tablet being better than SCIT]	−9.3 (−15.8;−2.3) [99%]	−8.0 (−16.3;−0.2) [97%]	−8.9 (−17.7;−1.2) [99%]

Abbreviations: CSMS, combined symptom‐medication score; SCIT, subcutaneous immunotherapy; SLIT‐tablet, sublingual AIT exclusively by tablets; VAS global allergy symptoms, visual analogue scale assessing the overall impact of allergic rhinitis symptoms on the user on that day; VAS Work, visual analogue scale assessing the work impact of allergic rhinitis symptoms on the user on that day.

By comparison with no AIT, SLIT‐tablet was associated with lower VAS Global allergy symptoms (−7.5, −12.1;−2.8), lower VAS Work (−5.0, −8.5;−1.5), and a lower CSMS (−3.7, −9.3;2.2). The probability of SLIT‐tablet being better than no AIT was 99% for both VAS Global allergy symptoms and VAS Work, and 89% for the CSMS.

By comparison with SCIT, SLIT‐tablet was also associated with lower VAS Global allergy symptoms (−10.2, −17.2;−2.8), lower VAS Work (−7.8, −15.1;0.2), and a lower CSMS (−9.3, −18.5;0.2). We observed a probability higher than 95% of SLIT‐tablet being associated with lower VASs and CSMS when compared to SCIT.

Similar results were observed in multivariable models adjusting for patients' sex, age and allergic comorbidities (Table 3). Tables 4 and 5 display the results for sensitivity analyses, with results stratified by country group and pollen season. Similar results were observed in separate analyses for countries where more observations were registered for SCIT when compared to those where more observations were registered for SLIT‐tablet. On the other hand, we observed larger differences for AIT versus no AIT when comparing days outside pollen seasons to those during pollen seasons.

**TABLE 4:** Results of sensitivity analyses for the comparisons between different grass immunotherapy (allergen immunotherapy [AIT]) types obtained with hierarchical models adjusting for the season, country and patient

	Difference in VAS global allergy symptoms	Difference in VAS work	Difference in CSMS
A. Hierarchical models for countries where there are more observations of SCIT than of SLIT‐tablet			
SCIT versus no AIT—Mean (CrI) [probability of SCIT being better than no AIT]	−0.6 (−4.0;2.7) [62%]	0.3 (−2.6;3.2) [41%]	0.3 (−3.3;4.2) [45%]
SLIT‐tablet versus no AIT—Mean (CrI) [probability of SLIT‐tablet being better than no AIT]	−9.3 (−18.9;−0.6) [98%]	−9.3 (−18.0;−0.4) [98%]	−14.2 (−24.6;−3.3) [99%]
SLIT‐tablet versus SCIT—Mean (CrI) [probability of SLIT‐tablet being better than SCIT]	−9.1 (−19.9;0.9) [96%]	−8.5 (−18.4;1.6) [95%]	−10.5 (−22.6;2.8) [94%]

B. Hierarchical models for countries where there are more observations of SLIT‐tablet than of SCIT			
SCIT versus no AIT—Mean (CrI) [probability of SCIT being better than no AIT]	6.8 (−3.8;17.0) [9%]	10.7 (0.4;19.8) [2%]	4.9 (−7.0;17.5) [20%]
SLIT‐tablet versus no AIT—Mean (CrI) [probability of SLIT‐tablet being better than no AIT]	−7.0 (−12.3;−1.0) [98%]	−3.7 (−7.5;0.6) [95%]	−1.1 (−7.9;6.2) [61%]
SLIT‐tablet versus SCIT—Mean (CrI) [probability of SLIT‐tablet being better than SCIT]	−12.5 (−23.3;−2.7) [99%]	−14.7 (−32.5;0.3) [97%]	−5.6 (−17.9;8.7) [78%]

C. Days during pollen seasons			
SCIT versus no AIT—Mean (CrI) [probability of SCIT being better than no AIT]	0.9 (−3.1;5.1) [33%]	0.8 (−3.1;4.5) [33%]	1.4 (−3.4;6.1) [29%]
SLIT‐tablet versus no AIT—Mean (CrI) [probability of SLIT‐tablet being better than no AIT]	−0.3 (−6.9;6.2) [52%]	0.5 (−4.6;5.8) [42%]	1.0 (−5.9;8.5) [40%]
SLIT‐tablet versus SCIT—Mean (CrI) [probability of SLIT‐tablet being better than SCIT]	−3.6 (−13.1;5.3) [78%]	0.5 (−7.3;7.8) [44%]	−3.0 (−13.4;6.9) [72%]

D. Days outside pollen seasons			
SCIT versus no AIT—Mean (CrI) [probability of SCIT being better than no AIT]	−2.4 (−5.5;1.0) [91%]	−1.0 (−4.1;2.1) [73%]	−1.1 (−4.8;2.8) [69%]
SLIT‐tablet versus no AIT—Mean (CrI) [probability of SLIT‐tablet being better than no AIT]	−11.8 (−16.5;−7.0) [99%]	−6.2 (−9.7;−2.1) [99%]	−8.3 (−13.8;−2.9) [99%]
SLIT‐tablet versus SCIT—Mean (CrI) [probability of SLIT‐tablet being better than SCIT]	−13.7 (−23.0;−4.4) [99%]	−9.0 (−18.0;−0.4) [98%]	−9.9 (−19.2;−0.4) [98%]

Abbreviations: CSMS, combined symptom‐medication score; SCIT, subcutaneous immunotherapy; SLIT‐tablet, sublingual AIT exclusively by tablets; VAS global allergy symptoms, visual analogue scale assessing the overall impact of allergic rhinitis symptoms on the user on that day; VAS work, visual analogue scale assessing the work impact of allergic rhinitis symptoms on the user on that day.

**TABLE 5:** Results of sensitivity analyses for the comparisons between different grass immunotherapy (allergen immunotherapy [AIT]) types obtained with hierarchical models adjusting for the season, country, patient and his/her characteristics (sex, age and comorbidities)

	Difference in VAS global allergy symptoms	Difference in VAS work	Difference in CSMS
A. Hierarchical models for countries where there are more observations of SCIT than of SLIT‐tablet			
SCIT versus no AIT—Mean (CrI) [probability of SCIT being better than no AIT]	0.01 (−3.1;3.2) [49%]	0.7 (−2.0;3.8) [31%]	0.8 (−3.1;4.8) [34%]
SLIT‐tablet versus no AIT—Mean (CrI) [probability of SLIT‐tablet being better than no AIT]	−9.5 (−20.1;0.1) [97%]	−7.5 (−16.8;1.6) [95%]	−10.7 (−21.4;0.3) [97%]
SLIT‐tablet versus SCIT—Mean (CrI) [probability of SLIT‐tablet being better than SCIT]	−9.7 (−19.6;1.0) [96%]	−10.2 (−21.0;0.5) [97%]	−8.0 (−20.5;3.5) [91%]

B. Hierarchical models for countries where there are more observations of SLIT‐tablet than of SCIT			
SCIT versus no AIT—Mean (CrI) [probability of SCIT being better than no AIT]	7.9 (−3.5;18.0) [8%]	11.4 (2.1;21.1) [1%]	6.8 (−5.6;18.6) [14%]
SLIT‐tablet versus no AIT—Mean (CrI) [probability of SLIT‐tablet being better than no AIT]	−6.1 (−12.0;−0.8) [98%]	−3.2 (−7.4;1.1) [92%]	−0.6 (−7.7;5.6) [55%]
SLIT‐tablet versus SCIT—Mean (CrI) [probability of SLIT‐tablet being better than SCIT]	−14.3 (−23.6;−5.9) [99%]	−15.2 (−25.2;−5.0) [99%]	−5.3 (−20.0;6.9) [73%]

C. Days during pollen seasons			
SCIT versus no AIT—Mean (CrI) [probability of SCIT being better than no AIT]	0.7 (−3.5;4.8) [37%]	0.7 (−3.1;4.5) [31%]	0.7 (−4.3;5.5) [38%]
SLIT‐tablet versus no AIT—Mean (CrI) [probability of SLIT‐tablet being better than no AIT]	−0.5 (−6.9;6.1) [57%]	0.3 (−5.0;5.7) [45%]	1.2 (−6.4;8.6) [37%]
SLIT‐tablet versus SCIT—Mean (CrI) [probability of SLIT‐tablet being better than SCIT]	−3.8 (−12.9;5.0) [78%]	−0.2 (−7.7;7.1) [51%]	−1.2 (−13.2;8.8) [56%]

D. Days outside pollen seasons			
SCIT versus no AIT—Mean (CrI) [probability of SCIT being better than no AIT]	−1.7 (−4.7;1.3) [85%]	−0.6 (−3.3;2.2) [65%]	−0.4 (−4.8;3.5) [56%]
SLIT‐tablet versus no AIT—Mean (CrI) [probability of SLIT‐tablet being better than no AIT]	−11.7 (−16.4;−7.4) [99%]	−5.5 (−9.2;−1.9) [99%]	−6.3 (−12.3;−0.2) [97%]
SLIT‐tablet versus SCIT—Mean (CrI) [probability of SLIT‐tablet being better than SCIT]	−10.7 (−19.1;−3.0) [99%]	−11.2 (−19.8;−3.5) [99%]	−9.9 (−18.8;0.9) [96%]

Abbreviations: CSMS, combined symptom‐medication score; SCIT, subcutaneous immunotherapy; SLIT‐tablet, sublingual AIT exclusively by tablets; VAS global allergy symptoms, visual analogue scale assessing the overall impact of allergic rhinitis symptoms on the user on that day; VAS work, visual analogue scale assessing the work impact of allergic rhinitis symptoms on the user on that day.

## 4 DISCUSSION

There is a clear unmet need for the further development and evaluation of validated tools to investigate the clinical efficacy of AIT under a real‐life scenario complementary to RCTs.^27^, ^28^ This study complements a recent proof‐of‐concept study.^20^ It is unique and demonstrates that, in grass pollen allergy patients, SLIT‐tablet is associated with lower VAS Global allergy symptoms, lower VAS Work and lower CSMS when compared to SCIT or no AIT. Subcutaneous allergen immunotherapy and no AIT showed similar levels for all three outcomes.

### 4.1 Strengths and limitations

This study has important strengths. Real‐world data from a large set of users from 10 different European countries have been assessed in this analysis. In addition, observations were clustered by users, country and season, thus taking into account certain potential individual confounders. Finally, MASK‐air^®^ VAS Global allergy symptoms and VAS Work have revealed high intra‐rater validity and moderate‐high validity, test‐retest reliability and responsiveness.^24^

This study also has some further limitations. First, there is the possibility of misclassification, given that the identification of patients with grass allergy and under each AIT type was based on information provided by the patients themselves and not on standardised diagnostics and physician evaluation as recommended in guidelines.^29^, ^30^ Such misclassification may lead to an underreporting of both grass allergy and AIT use. To partly account for this, as well as for a longer‐lasting effect of AIT, we included all days of the patients reporting to be under AIT, irrespective of the specific days on which AIT was actually used.

Second, selection biases are known to exist in mHealth. MASK‐air^®^ users are not representative of the general population of allergic rhinitis patients with grass allergy, with an overrepresentation of users suffering from more severe disease and/or receiving more specialised treatment (which would explain why VAS values tend to be higher on the first day of reporting than on subsequent days).^21^, ^22^ In addition, it is possible to hypothesise that days with patients feeling worse tend to be more frequently reported than those with patients experiencing no or mild symptoms, although this probably occurs in a non‐differential way, irrespective of the AIT type under which the patients may be. However, a differential reporting bias may result from the fact that SLIT‐tablet is taken every day, self‐administered by the patient, in contrast to SCIT, which is given every 4–6 weeks. On the other hand, as the MASK‐air^®^ app is to be filled in every day, with each question concerning that specific day, recall bias may not have a substantial impact on our results.

Third, we did not follow a product‐specific approach for each AIT application route (as recommended in current international guidelines on AIT^29^, ^30^), but analysed differences between routes of administration in a generic way. Moreover, we did not report on SLIT‐drops because there was only one country with more than 150 reported days of SLIT‐drops and SLIT‐tablet use (France), and only one country with more than 150 reported days of both SLIT‐drops and SCIT (Lithuania). In this country, the mean number of recorded days per user was substantially higher than that observed in other countries.

Fourth, we used, as previously, a cross‐sectional design.^20^, ^21^, ^22^ When we launched MASK‐air^®^, it was expected that patients would use the app regularly and that it would be possible to perform a longitudinal analysis. However, patients use the app for short periods of time (in this study, a mean number of 39 days were reported per user) and intermittently. Analyses for intermittent use (consecutive and non‐consecutive data) have been performed.^21^ This cross‐sectional approach has been shown to be effective in raising new hypotheses, subsequently confirmed by epidemiologic studies.^31^, ^32^, ^33^ However, the cross‐sectional approach of this study precludes the establishment of a causal relationship between AIT use and reported symptoms, as well as the assessment of AIT adherence and therapy duration. Measurement of the latter variables would be particularly relevant given (i) the possible differences in the time needed for SLIT and SCIT to become effective, and (ii) the fact that SCIT may ensue more physician‐patient interaction (as, contrary to SLIT, it cannot be self‐administered by the patient at home). If patients tend to report symptoms more often when feeling worse or after establishing care with an allergist, this could imply a bias in the estimation of SLIT and SCIT efficacy (particularly in the first months after AIT initiation). Solutions in addressing these methodological biases of real‐world evidence in AIT need to be elaborated in the future.

### 4.2 Findings

While symptom levels are low, suggesting that AIT patients are not severe, a previous study has found median VAS levels of around 50/100 on the first day of reporting (when considering the pollen season).^21^ The low VAS levels reported in most observations may at least partly reflect the efficacy of medications. Moreover, we reported days under allergen exposure and days without. A new study has been scheduled to include pollen counts rather than simply accounting for the pollen season.

Taking into account the selection of patients and the nature of observational mHealth studies, this study suggests that grass pollen allergic patients under SLIT‐tablet report less severe symptoms than those under no AIT. Several hypotheses can be postulated to explain this finding, including (i) real differences in the efficacy or effectiveness of AIT, (ii) differences in the mode of administration for each AIT type (with SLIT‐tablet, contrary to SCIT, being administered at home and on a daily basis), (iii) differences in the time needed for the various types of AIT to achieve effectiveness, (iv) differences on the baseline symptoms of patients, and (v) the greater diversity of SCIT products compared to SLIT formulations (which may also explain the heterogeneity observed in the meta‐analyses of SCIT trials).^34^, ^35^ Therefore, conclusions cannot be made on the efficacy or effectiveness of AIT, as several limitations exist (as outlined above). For instance, users under AIT tend to be more closely followed by their physicians and demonstrate a higher adherence to physicians' recommendations (the so‐called ‘Hawthorne effect’ as one component of unspecific treatment effects in AIT^34^). This study prompts the need for future observational prospective studies, combining patients' and physicians' inputs and adjusting for the most relevant confounders.

The effect of AIT on work was previously suggested by two studies using the MASK‐air^®^ approach.^19^, ^36^ In this study, we have confirmed that SLIT‐tablet can be associated with improved VAS Work. Once further longitudinal studies have been conducted, this will open the door to ascribing a monetary value to this form of treatment and to performing subsequent economic evaluation studies.

We have used a recently validated CSMS.^37^ Differences between SLIT‐tablet, SCIT or no AIT were smaller when estimated in relation to the CSMS than in relation to VAS Global allergy symptoms or VAS Work. It is possible that patients under SLIT‐tablet have a reduced treatment use which might explain the difference.

### 4.3 Generalisability

The study was carried out in nine European countries and can be extended to the whole of Europe. However, it does not necessarily apply to countries where AIT is used with different allergen extracts and regimens.

## 5 CONCLUSION

When compared to no AIT and to SCIT, SLIT was found to be associated with a better allergic rhinitis symptom control, impact on work and CSMS in patients with grass pollen allergy. By contrast, no such differences were observed when comparing SCIT to no AIT. Following these results, future longitudinal studies are needed (taking potentially relevant confounders into account and following internationally harmonised standards for performing and reporting real‐world data in AIT) to assess the effectiveness in improving allergic rhinitis control.

## CONFLICT OF INTEREST

IA reports personal fees from Hikma, Roxall, Astra Zeneca, Menarini, UCB, Faes Farma, Sanofi, Mundipharma, Bial, Amgen, Stallergenes Greer, Bayer. JB reports personal fees from Chiesi, Cipla, Hikma, Menarini, Mundipharma, Mylan, Novartis, Sanofi‐Aventis, Takeda, Teva, Uriach, other from KYomed‐Innov, personal fees from Purina, TC reports grants from Stallergenes Greer. AC reports personal fees from GSK, AstraZeneca, Sanofi, Novartis, Boehringer Ingelheim, Mylan, Mantecorp, Eurofarma. PhD reports personal fees and non‐financial support from Alk Abello, Stallergenes Greer. TH reports personal fees from GSK, Mundipharma, Orion Pharma, and Sanofi. JCI reports personal fees from Laboratorios Casasco, Abbott Ecuador, Bago Bolivia, Sanofi, Eurofarma Argentina, VK reports other from BerlinCHemie Menarini, other from Norameda. DLL reports personal fees from Allakos, Amstrong, Astrazeneca, DBV Technologies, Grunenthal, GSK, Mylan/Viatris, Menarini, MSD, Novartis, Pfizer, Sanofi, Siegfried, UCB, Alakos, Gossamer, Carnot, grants from Sanofi, Astrazeneca, Novartis, Circassia, UCB, GSK, Purina institute. RL reports grants and personal fees from GSK, AZ, Chiesi, Novartis, personal fees from Sanofi. MM reports personal fees from Menarini, personal fees from Astra Zeneca, personal fees from GSK, personal fees from Mylan, personal fees from Sanofi, personal fees from Pfizer, personal fees from Chiesi, RM reports personal fees from ALK, allergopharma, Allergy Therapeutics, Friulchem, Hexal, Servier, Klosterfrau, Bayer, FAES, GSK, MSD, Johnson&Johnson, Meda, Stada, UCB, Nuvo, Menarini, Mundipharma, Pohl‐Boskamp,grants from ASIT biotech, Leti, Optima, BitopAG, Hulka, Ursapharm, Inmunotek, grants and personal fees from Bencard, Stallergenes, grants, personal fees and non‐financial support from Lofarma, non‐financial support from Roxall, Atmos, Bionorica, Otonomy, Ferrero, personal fees and non‐financial support from Novartis. JM reports personal fees and other from SANOFI‐GENZYME & REGENERON, NOVARTIS, ALLAKOS, grants and personal fees from MYLAN Pharma, URIACH Group, personal fees from Mitsubishi‐Tanabe, Menarini, UCB, AstraZeneca, GSK, MSD. NGP reports personal fees from Novartis, Nutricia, HAL, MENARINI/FAES FARMA, SANOFI, MYLAN/MEDA, BIOMAY, AstraZeneca, GSK, MSD, ASIT BIOTECH, Boehringer Ingelheim, grants from Gerolymatos International SA, Capricare. OP reports grants and personal fees from ALK‐Abelló, Allergopharma, g Stallergenes Greer, HAL Allergy Holding B.V./HAL Allergie GmbH, Bencard Allergie GmbH/Allergy Therapeutics, Lofarma, grants and personal fees from ASIT Biotech Tools S.A., Laboratorios LETI/LETI Pharma, Anergis S.A., GlaxoSmithKline, personal fees from Astellas Pharma Global, personal fees from EUFOREA, ROXALL Medizin, Novartis, Sanofi‐Aventis and Sanofi‐Genzyme, Med Update Europe GmbH, streamedup! GmbH, Mobile Chamber Experts (a GA2LEN Partner), Indoor Biotechnologies, MEDA Pharma/MYLAN, John Wiley and Sons, AS, Paul‐Martini‐Stiftung (PMS), Ingress‐Health HWM, Regeneron Pharmaceuticals Inc., grants from Pohl‐Boskamp, Inmunotek S.L., Biomay, Circassia. JS reports grants and personal fees from SANOFI, personal fees from GSK, NOVARTIS, ASTRA ZENECA, MUNDIPHARMA, FAES FARMA. AMTB reports grants and personal fees from Novartis, Mundipharma, Teva Pharma, GSK (GlaxoSmithKline), AstraZeneca, grants from Leti, personal fees from BIAL. Dr. Tsiligianni reports personal fees from Honoraria for educational activities, speaking engagements, advisory boards from Boehringer Ingelheim, Astra Zeneca, GSK, Novartis, MSD and grants from GSK Hellas Astra Zeneca, and Elpen. DW reports personal fees from ALK.

## AUTHOR CONTRIBUTIONS

BSP participated in methodology, formal analysis and writing ‐ original draft. OP participated in conceptualisation, formal analysis, supervision and writing ‐ original draft. LFA participated in methodology and writing ‐ review & editing. JAF participated in conceptualisation, formal analysis, supervision and writing ‐ review & editing. JB participated in conceptualisation, formal analysis, supervision and writing ‐ original draft. All remaining authors participated in data collection and writing ‐ review & editing.

## Supporting information

Supplementary Material S1
