# Supplementary material for: Allergen immunotherapy in MASK‐air users in real‐life: Results of a Bayesian mixed‐effects model
Source: Clin Transl Allergy. 2022 Mar 28;12(3):e12128. doi: 10.1002/clt2.12128 (PMC8967259; doi:10.1002/clt2.12128)
Supplement: Supplementary file 1 — Supplementary Material S1 [file CLT2-12-e12128-s001.docx]

**Supplementary Tables**

**Supplementary Table 1. Values which multiply by the visual analogue scale (VAS) on allergic rhinitis global allergy symptoms so as to compute the combined symptom-medication score.**

| **Medication** | **VAS<50** | **50≤VAS<75** | **VAS>75** |
| --- | --- | --- | --- |
| No medication | 1.00 | 1.00 | 1.00 |
| Oral or intranasal or ocular H1-antihistamines | 1.10 | 1.05 | 1.00 |
| Intranasal steroids | 1.40 | 1.20 | 1.10 |
| Intranasal steroids + oral or ocular H1-antihistamines | 1.50 | 1.25 | 1.12 |
| Intranasal steroids + intranasal H1-antihistamines | 2.00 | 1.50 | 1.25 |
| Intranasal steroids + intranasal H1-antihistamines + oral/ocular H1-antihistamines | 2.10 | 1.55 | 1.30 |
| Oral steroids | 2.00 | 1.40 | 1.20 |
| Other rhinitis medication | 1.10 | 1.05 | 1.00 |

**Supplementary Table 2. Visual analogue scales (VASs) used for daily monitoring the impact of allergic rhinitis and asthma symptoms in MASK-air®**

| **Scale** | **Question** |
| --- | --- |
| VAS Global allergy symptoms | “Overall how much are your allergic symptoms bothering you today?” |
| VAS Nose | “How much are your nose symptoms bothering you today?” |
| VAS Eyes | “How much are your eyes symptoms bothering you today?” |
| VAS Asthma | “How much are your asthma symptoms bothering you today?” |
| VAS Work | “How much are your allergic symptoms affecting your work today?” |
| VAS Sleep | “How did you sleep last night?” |

English version of the questions. These questions are available in 29 different languages.
